# Activación de la microglía en el hipocampo asociada con lesión del nervio facial

**DOI:** 10.7705/biomedica.6216

**Published:** 2022-03-01

**Authors:** Jeimmy Cerón, Julieta Troncoso

**Affiliations:** 1 Laboratorio de Neurofisiología Comportamental, Departamento de Ciencias Fisiológicas, Facultad de Medicina, Universidad Nacional de Colombia, Bogotá, D.C., Colombia Universidad Nacional de Colombia Universidad Nacional de Colombia Bogotá, D.C. Colombia; 2 Departamento de Biología, Facultad de Ciencias, Universidad Nacional de Colombia, Bogotá, D.C., Colombia Universidad Nacional de Colombia Universidad Nacional de Colombia Bogotá, D.C. Colombia

**Keywords:** nervio facial, hipocampo, inmunohistoquímica, ratas., Facial nerve, hippocampus, immunohistochemistry, rats.

## Abstract

**Introducción.:**

Las lesiones del nervio facial afectan la plasticidad a largo plazo en el hipocampo, así como la memoria de reconocimiento de objetos y la memoria espacial, dos procesos dependientes de esta estructura. Si bien se ha descrito una activación de la microglía en la corteza motora primaria asociada con esta lesión, no se conoce si ocurre algo similar en el hipocampo.

**Objetivo.:**

Caracterizar en ratas el efecto de la lesión unilateral del nervio facial sobre la activación de células de la microglía en el hipocampo contralateral.

**Materiales y métodos.:**

Se hicieron experimentos de inmunohistoquímica para detectar células de la microglía en el hipocampo de ratas sometidas a lesión irreversible del nervio facial. Los animales se sacrificaron en distintos momentos después de la lesión, para evaluar la evolución de la proliferación (densidad de células) y la activación (área celular) de la microglía en el tejido del hipocampo. Los tejidos cerebrales de los animales de control se compararon con los de animales lesionados sacrificados en los días 1,3, 7, 21 y 35 después de la lesión.

**Resultados.:**

Las células de la microglía en el hipocampo de animales con lesión del nervio facial mostraron signos de proliferación y activación a los 3, 7 y 21 días después de la lesión. Sin embargo, al cabo de cinco semanas, estas modificaciones se revirtieron, a pesar de que no hubo recuperación funcional de la parálisis facial.

**Conclusiones.:**

La lesión irreversible del nervio facial produce proliferación y activación temprana y transitoria de las células de la microglía en el hipocampo. Estos cambios podrían estar asociados con las modificaciones electrofisiológicas y las alteraciones comportamentales dependientes del hipocampo descritas recientemente.

## Abstract (en)

**Introduction:**

Facial nerve injury induces changes in hippocampal long-term synaptic plasticity and affects both object recognition memory and spatial memory consolidation (i.e., hippocampus-dependent tasks). Although facial nerve injury-associated microglíal activation has been described regarding the primary motor cortex, it has not been ascertained whether something similar occurs in the hippocampus. Peripheral nerve injury- associated microglíal changes in hippocampal tissue could explain neuronal changes in the contralateral hippocampus.

**Objective:**

To characterize the effect of unilateral facial nerve injury on microglíal proliferation and activation in the contralateral hippocampus.

**Materials and methods:**

. Immunohistochemical experiments detected microglíal cells in the hippocampal tissue of rats that had undergone facial nerve injury. The animals were sacrificed at specific times after injury to evaluate hippocampal microglíal cell proliferation (cell density) and activation (cell area); sham-operated animals were compared to lesioned animals sacrificed 1,3, 7, 21, or 35 days after injury.

**Results:**

Facial nerve-injured rats’ hippocampal microglíal cells proliferated and adopted an activated phenotype 3- to 21-days post-lesion. Such modifications were transient since the microglíal cells returned to their resting state five weeks after injury, despite the injury’s irreversible nature.

**Conclusions:**

Facial nerve injury causes the transient proliferation and activation of microglíal cells in the hippocampus. This finding might partly explain the morphological and electrophysiological changes described for CA1 pyramidal neurons and the impairment of spatial memory consolidation which has previously been observed in facial nerve-injured rats.

El Grupo de Investigación de Neurofisiología Comportamental de la Universidad Nacional intenta dilucidar, desde hace ya más de una década, los cambios electrofisiológicos, moleculares y morfológicos que ocurren en el sistema nervioso central cuando hay una lesión de nervio periférico. Para ello, utiliza el modelo de lesión del nervio facial en roedores, por su idoneidad y practicidad ^1^.

Hace unos años se constató que aparecen modificaciones morfológicas y electrofisiológicas en la *corteza motora primaria de las vibrisas (Vibrisal Motor Cortex, vM1*), cuando hay lesión del nervio facial ^2^^,^^3^. Asimismo, se caracterizó la activación de la microglía que circunda la *corteza motora primaria de las vibrisas*, asociada con dicha lesión ^4^.

Recientemente, se observó que tras lesiones del nervio facial se producen modificaciones que se extienden más allá de las áreas del cerebro directamente relacionadas con el procesamiento sensitivo-motor en la corteza. Específicamente, se ha establecido que la lesión del nervio facial conduce a una disminución en la potenciación a largo plazo de las sinapsis en el hipocampo de CA3 a CA1 ^5^. Estas modificaciones en la plasticidad del hipocampo se añaden a hallazgos previos que indican que los animales con lesión del nervio facial presentan dificultades para la consolidación de memorias espaciales y, además, presentan elevados niveles de corticosterona plasmática, comparados con los animales de control ^6^^,^^7^. Además, en estudios recientes se encontró una reducción significativa de la complejidad de los árboles dendríticos de las células piramidales de CA1 y CA3, así como de las espinas dendríticas en esas células, asociadas con la lesión del nervio facial (Troncoso J. Deterioro de la memoria espacial y modificaciones funcionales y estructurales en el hipocampo de ratas sometidas a lesión del nervio facial. Memorias, XII Congreso Nacional - XIII Seminario Internacional de Neurociencias 2021. https://colne.org.co/congreso/#speakers).

Las células de la microglía se han considerado tradicionalmente como los macrófagos del sistema nervioso central ^8^. En reposo, son células ramificadas que, ante una señal inmunológica provocada por daños tisulares, se activan acortando sus procesos, aumentando el número de ramificaciones y adoptando una forma ameboide fagocítica ^9^^-^^11^. Además de su función inmunológica, se sabe que cumplen un papel importante en el control de la función sináptica, manteniendo su integridad ^12^^-^^15^. Las células de la microglía son extremadamente sensibles a perturbaciones producidas por una gran cantidad de variables. Por ejemplo, se sabe que el estrés puede alterar la estructura y, por lo tanto, la función de las células de la microglía ^16^^-^^18^. En ese sentido, se ha descrito que las hormonas relacionadas con el estrés (la corticosterona y la norepinefrina, entre otras) producen un cambio estructural de las células de la microglía y que estas “remodelaciones” pueden afectar la arquitectura y la función neuronal ^19^.

En este contexto, el objetivo del presente estudio fue conocer la dinámica de la activación y proliferación de las células de la microglía que rodean las neuronas del hipocampo después de lesiones irreversibles del nervio facial. Estos cambios podrían explicar, al menos en parte, las modificaciones estructurales y funcionales observadas en esta estructura, así como los impedimentos en tareas de memoria dependientes del hipocampo, tras la lesión.

## Materiales y métodos

### Sujetos experimentales

Se usaron 24 ratas albinas macho (*Ratus norvegicus*) de la cepa Wistar, de 290 ± 20 g, procedentes del Bioterio del Instituto Nacional de Salud. Los animales se alojaron en grupos de cuatro en cajas de policarbonato (38 x 32 x 18 cm) que contenían una capa de aserrín en el fondo, la cual se cambiaba cada tercer día. Durante el tiempo en que permanecieron en el Laboratorio de Neurociencias de la Universidad Nacional, los animales se mantuvieron con humedad (40 ± 5 %) y temperatura (20 ± 3 °C) controladas, con ciclos de 12 horas de luz y oscuridad (luces encendidas a las 7:00 am), y libre acceso a agua y comida.

### Diseño experimental

**Figura 1:**
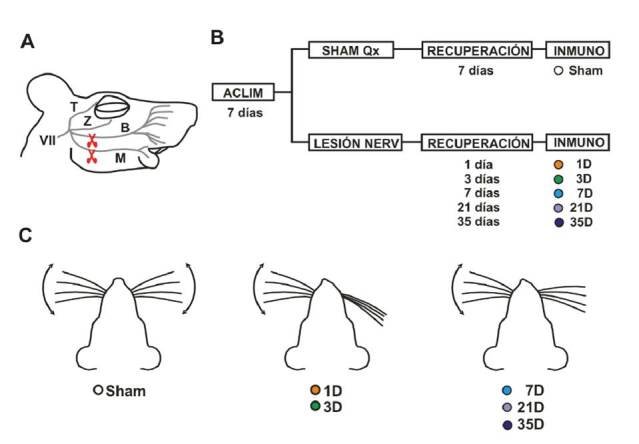
Metodología, diseño experimental y efectos de la lesión en la función motora. **A.** Esquema del nervio facial en la rata. Al entrar en la cara del animal, el nervio facial (Vil par craneal) se divide en las ramas temporal (T), zigomática (Z), bucal (B) y mandibular (M); la lesión irreversible del nervio facial consiste en el corte de las ramas B y M (tijeras). **B.** Diseño experimental. Los animales se dividieron aleatoriamente en dos grupos: de falsa cirugía de lesión (*sham*, de control) y con lesión irreversible del nervio facial. Los animales lesionados, a su vez, se subdividieron en 5 grupos: 1D, 3D, 7D, 21D y 35D (que se sacrificaron en los días 1,3, 7, 21 y 35 de la lesión). Los cerebros de los animales de los dos grupos se procesaron para tinción inmunohistoquímica con el anticuerpo anti-lba-1, con el fin de detectar células de la microglía. **C.** Evaluación de la función motora. La correcta lesión del nervio facial se corroboró por la ausencia del movimiento activo de las vibrisas del lado lesionado. En los primeros días posteriores a la lesión, las vibrisas se paralizaron y aparecían completamente hacia atrás; una semana después de la lesión, los animales recuperaron tonicidad en las vibrisas del lado lesionado, pero continuaron con parálisis facial durante por lo menos 5 semanas después de lesión.

Los 24 animales experimentales se dividieron aleatoriamente en dos grandes grupos: uno de control (*sham*), con falsa cirugía de lesión (n=4), y uno con lesión irreversible del nervio facial (n=20) (figura 1 A). Los animales del grupo con lesión irreversible se subdividieron a su vez en: 1D, 3D, 7D, 21D y 35 D, los cuales se sacrificaron en los días 1,3, 7, 21 y 35 después de la lesión, respectivamente (n=4 cada uno). Los animales del grupo de control se sacrificaron después de una semana de la falsa cirugía de lesión. Un esquema del diseño experimental se muestra en la figura 1B.

Aclim: aclimatación por 7 días; Qx: cirugía; Inmuno: inmunohistoquímica

### Preparaciones experimentales

*Lesión irreversible de nervio facia*l. Al entrar a la cara, el tronco del nervio facial se divide en múltiples ramas (temporal, cigomática, bucal y mandibular) (figura 1 A). La lesión irreversible del nervio facial se produjo cortando las ramas mandibular y bucal (M y B, figura 1 A). Estas ramas son las que inervan la musculatura intrínseca de las vibrisas, cuya contracción promueve el movimiento activo de estos pelos sensoriales.

Para el procedimiento, los animales recibieron anestesia general (ketamina, 100 mg/kg, más xilazina, 10 mg/kg) y se colocaron en decúbito lateral para afeitar la región preauricular del lado derecho. Se hizo una incisión y luego una disección roma por planos hasta aislar las ramas del nervio facial. Las ramas mandibular y bucal se detectaron mediante estimulación eléctrica y se cortaron retirando un segmento de 1 mm de cada una de ellas. La efectividad de la lesión se confirmó por estimulación eléctrica de las regiones proximales y distales de cada rama lesionada. Los bordes de piel se suturaron con puntos discontinuos utilizando seda 4-0.

*Falsa cirugía de* lesión (grupo de control, *sham*). Los animales se anestesiaron con 100 mg/kg de ketamina más 10 mg/kg de xilazina, se colocaron en decúbito lateral para afeitar la región preauricular del lado derecho y se les hizo una incisión horizontal de un cm por encima del ángulo mandibular derecho. Se disecaron las ramas bucal y mandibular del nervio facial, manteniendo intacta su integridad. La incisión en la piel se suturó con puntos discontinuos utilizando seda 4-0.

*Inmunohistoquímica*. El procedimiento de inmunohistoquímica se hizo según lo descrito por Moreno, *et al*. ^6^, después de anestesiar profundamente a los animales con uretano (1,6 g/kg, intraperitoneal). Se abrió la cavidad torácica para exponer el corazón y se insertó una cánula en el ventrículo izquierdo, con el fin de desangrar al animal mediante perfusión de solución salina al 0,9 % y fijación del tejido cerebral en paraformaldehído al 4 % preparado en 0,1 M de PBS (pH 7,4). Los cerebros se almacenaron a 4 °C en solución fijadora (paraformaldehído al 4 % preparada en 0,1 M de solución tampón PBS) durante 48 horas y luego se transfirieron a una solución de sacarosa al 30 % para su crioprotección. Se obtuvieron cortes coronales de 50 pm de grosor utilizando un criostato a una temperatura de -20 °C. Se recolectaron y almacenaron secciones de tejido cerebral de cada sujeto para un total de cuatro series que comprendían el hipocampo dorsal (coordenadas desde el bregma, AP: -1,5 a -4,3 mm) ^20^.

La inmunorreactividad de la proteína Iba1 (Ionized calcium binding adaptor molecule 1), que se expresa de forma específica en macrófagos y microglía ^21^, se detectó empleando antisuero policlonal producido en conejo: anti-lba1 (Wako, Cat. #019-19741). La unión antígeno-anticuerpo se reveló utilizando el estuche ABC (avidina-biotina peroxidasa) de Vectastain™ (Laboratorios Vector, USA, Ref. PK 6101).

Antes del procedimiento, se hicieron lavados para retirar el anticongelante (2x10 minutos, 0,1 M de PB). Posteriormente, se bloqueó la peroxidasa endógena incubando los tejidos en H_2_0_2_ al 1 % durante 20 minutos para luego proceder a bloquear los sitios de unión inespecíficos con albúmina de suero bovino (BSA 1 %; Sigma, Ref. A7030-100G) y suero normal de cabra (1 %; Laboratorios Vector, USA, Ref. S-1000).

Las secciones se incubaron toda la noche con el anticuerpo primario (anti-lba1) (1:1.000 en la solución de bloqueo) a temperatura ambiente y en agitación. Posteriormente, se inició la incubación con el anticuerpo anticonejo secundario biotinilado producido en cabra (Laboratorios Vector, USA, Ref. PK 6101; 1:250, PB-Tritón, BSA al 0,3 %, suero normal de cabra al 0,3 %) y en agitación durante dos horas a temperatura ambiente.

A continuación, las secciones se incubaron con solución de avidina-biotina durante dos horas y se lavaron nuevamente en 0,1 M de PB para iniciar el proceso de revelado empleando una solución de 3,3’-diaminobenzidina (DAB 0,05 %, Laboratorios Vector, USA, Ref. SK-4100). Los cortes de tejido cerebral se montaron con pincel en portaobjetos previamente gelatinizados para evitar su despegue; una vez que los cortes se secaron por completo, se colocó el cubreobjetos (adherido mediante bálsamo de Canadá) para su posterior visualización en el microscopio de luz.

### Análisis de datos y pruebas estadísticas

El análisis de las células positivas para Iba1, tanto del conteo como del área, se sometieron a pruebas estadísticas de ANOVA de una vía para establecer diferencias significativas entre grupos. Las células positivas para lba-1, así como su área, se cuantificaron en las fotografías ampliadas de los cortes histológicos observados con microscopía óptica de luz con 400 aumentos, utilizando el programa Image J. Se evaluaron la densidad y el área de las células de la microglía de toda la formación del hipocampo, seleccionando aleatoriamente campos ópticos de cada una de las áreas estudiadas (CA1, CA3 y giro dentado).

Antes de los análisis, se calibró el programa configurando la equivalencia de mieras y pixeles mediante imágenes de una escala micrométrica. En las fotografías de interés, se ajustó la profundidad de color a 8 bits y se convirtieron en imágenes pseudobinarias por medio de la función *Threshold*, indicando posteriormente al programa los parámetros por medir. Las comparaciones planeadas se realizaron con el método Holm-Sidak. Se utilizó un valor crítico de significación de 0,05.

### Consideraciones éticas

Las condiciones de alojamiento, los procedimientos experimentales y la disposición de los residuos biológicos y químicos se hicieron observando las normativas nacionales e internacionales pertinentes: Resolución N° 008430 de 1993 del Ministerio de Salud y Ley 84 del 27 de diciembre de 1989, además de lo estipulado en la Resolución 2378 de 2008 del Ministerio de Protección Social.

Los experimentos fueron avalados por el Comité de Ética de la Facultad de Ciencias de la Universidad Nacional de Colombia. Se hizo un esfuerzo para reducir el número de animales utilizados y evitar su sufrimiento innecesario. También, se tuvieron en cuenta los procedimientos para el manejo y cuidado de animales de laboratorio recomendados por la normativa de la Unión Europea (8616091EU) y los Institutos Nacionales de Salud de los Estados Unidos de América (*National Research Council,* 2010).

## Resultados

Los animales sometidos a lesión de las ramas bucal y mandibular del nervio facial presentaron una parálisis facial del tercio inferior de la cara, tal y como se esperaba. La parálisis se evidenció por ausencia de movimientos activos de las vibrisas del lado lesionado, en comparación con los animales de control con lesión falsa (figura 1C). Durante la primera semana después de lesión, se evidenció en los animales una retracción completa de las vibrisas del lado lesionado (grupos 1D y 3D). Sin embargo, a partir de esa primera semana, las vibrisas recuperaron tonicidad, aunque continuaron paralizadas (grupos 7D, 21 D, 35D), ya que la lesión se realizó cortando y retirando una parte de cada rama del nervio facial, se impidió la reinervación y, por lo tanto, la posibilidad de la recuperación de la función motora (parálisis irreversible) (figura 1C).

**Figura 2:**
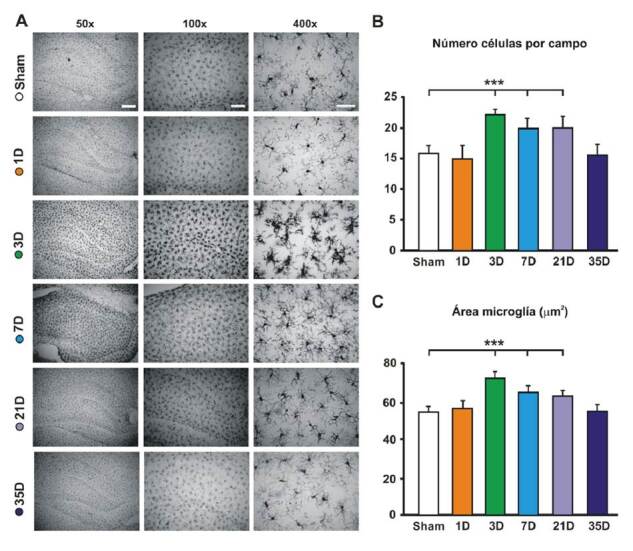
Modificación de las células de la microglía en el hipocampo asociada con la lesión del nervio facial. **A.** Evolución temporal de los cambios en la microglía hipocampal. Se muestran microfotografías representativas de cada grupo (*sham* y lesionados), sacrificados en distintos momentos después de la lesión, en las que se visualiza la inmunorreactividad frente a lba-1 en el tejido del hipocampo contralateral a la lesión. Aumentos: a la izquierda, 50X; al centro, 100X, y a la derecha, 400X. En las fotografias superiores (grupo de control, *sham*) se observa una microglía típica del reposo, con ramificaciones delgadas y cuerpos celulares pequeños, similares a las encontradas en animales sacrificados el día posterior a la lesión (1D). A los tres días, se evidenciaron una proliferación y activación de la microglía (3D), que se revirtieron a las cinco semanas de la lesión (35D). Barras de calibración, izquierda: 300 mm; centro: 150 mm; derecha: 50 mm. **B.** Densidad de células de la microglía. La cuantificación del número de células por campo evidenció una proliferación significativa de microglías a partir del tercer día de la lesión (3D), que se sostuvo hasta el día 21D. **C.** Activación de las células de la microglía. La cuantificación del área de los somas de las células de la microglía permitió estimar su activación. Se observa un aumento significativo del área de estas células en los días 3, 7 y 21 de la lesión (3D, 7D y 21D), que se revirtió a los 35 días de la lesión (35D).

La figura 2A corresponde a fotografías con distintos aumentos (50X, 100X y 400X) del hipocampo contralateral al lado lesionado de animales representativos de cada grupo experimental, comparados con los animales de control (*sham*). La inmunorreactividad de la proteína Iba1 del hipocampo de los animales con falsa cirugía de lesión, evidenció que las células de la microglía presentaban una morfología típica de células residentes en reposo, caracterizada por un cuerpo celular pequeño acompañado por procesos largos y ramificados (figura 2A, panel superior).

* p<0,001

Sin embargo, cuando se analizaron las células de la microglía del hipocampo de animales sacrificados en distintos momentos después de la axotomía del nervio facial, se observaron cambios evidentes tanto en su morfología como en su densidad numérica. Por una parte, es evidente que la lesión irreversible del nervio facial produjo una proliferación de las células de la microglía. El análisis de varianza arrojó diferencias significativas en el número de células positivas para lba-1 con respecto al grupo de control (F_(590)_=21,98; p<0,001). Específicamente, las comparaciones revelaron un mayor número de células de la microglía a los 3 días (*sham Vs*. 3D, t=8,28; p<0,001), a los 7 días (*sham Vs*. 7D, t=4,99; p<0,001) y a los 21 días (*sham Vs*. 21D, t=4,77; p<0,001) de la lesión (figura 2B).

Por otra parte, no se encontraron diferencias significativas en el número de células de la microglía en el hipocampo de los animales sacrificados en el día 1 (*sham Vs*. 1D, t=0,80; p=0,42),) ni en el día 35 (*sham Vs*. 35D, t=0,37; p=0,71) de la lesión. Esto indica que el aumento del número de células de la microglía en el hipocampo asociado con la lesión fue de aparición temprana, ya que se manifestó a partir del tercer día de la lesión. Además, esta proliferación es transitoria, ya que 5 semanas después de la lesión, hubo una recuperación en el patrón de distribución de las células de la microglía muy similar a la del grupo control, a pesar de que no se recuperó la función motora.

Por otro lado, fue evidente que la forma de las células de la microglía cambió con la axotomía del nervio facial: se presentaron alteraciones morfológicas de engrasamiento y retracción de sus ramificaciones, con cuerpos celulares agrandados evidentes a partir del tercer día de la lesión (figura 2A).

En el análisis de varianza, hubo diferencias significativas en el área de los somas de las células de la microglía tras la lesión, en comparación con el grupo de control (F_(5,426)_=276,59; p<0,001). Específicamente, se encontraron aumentos en el área somática microglíal de la formación del hipocampo de los animales sacrificados a los 3 días (*sham* Vs. 3D, t=28,51; p<0,001), a los 7 días (*sham Vs*. 7D, t=15,45; p<0,001) y a los 21 días (*sham Vs*. 21D, t=11,57; p<0,001) de la lesión (figura 2C).

Por otra parte, no se encontraron diferencias significativas en el área de las células de la microglía de los animales sacrificados en el día 1 (*sham Vs*. 1D, t=0,28; p=0,35), ni en el día 35 (*sham Vs* 35D, t=0,93; p=0,78) de la lesión, en comparación con el grupo de control. Ello indicaría que hubo una activación temprana de las células de la microglía, con agrandamiento del soma y reducción de los procesos, que se evidenció a partir de los 3 días de la lesión. Esta activación, sin embargo, fue transitoria, ya que la microglía retornó a sus niveles básales de activación y recobró características morfológicas similares a las de los animales sometidos a falsa cirugía a las 5 semanas de la axotomía.

## Discusión

En este estudio se caracterizó la evolución de la proliferación y activación de las células de la microglía del hipocampo asociadas con la lesión del nervio facial. Esta modificación fue de aparición temprana aunque transitoria, dado que se hizo evidente a los tres días de la lesión, pero se revirtió cinco semanas después, aunque no hubo recuperación de la función motora, dada la naturaleza irreversible de la lesión. En estudios previos, se había descrito una activación de la microglía similar en la corteza motora primaria de las vibrisas ^4^, asociada con modificaciones estructurales y electrofisiológicas de las células piramidales corticales que realizan conexiones monosinápticas directas con el núcleo del facial ^2^^,^^3^.

Hay abundante evidencia de que el hipocampo de los mamíferos cumple un papel fundamental en la navegación en ambientes familiares y novedosos, así como en el aprendizaje y la memoria de la ubicación de objetos o lugares en el espacio ^22^^-^^25^. Las ratas son animales nocturnos que dependen bastante de sus habilidades de navegación para explorar el ambiente; esta navegación requiere del uso activo de las vibrisas faciales, las cuales funcionan como órganos táctiles de gran sensibilidad para la exploración activa de objetos y texturas, así como la detección de la distancia a la que se encuentran los objetos ^26^. Hay evidencia de que el hipocampo recibe y procesa información somatosensorial adquirida mediante el movimiento activo de las vibrisas faciales en la rata ^27^.

En estudios previos, se ha demostrado que la lesión del nervio facial está relacionada con impedimentos en la consolidación de memorias dependientes del hipocampo (memoria de reconocimiento de objetos y memoria espacial) ^6^^,^^7^. Esos hallazgos, sumados a recientes descripciones que indican que la potenciación a largo plazo en el hipocampo de animales con lesión del nervio facial es significativamente menor que en los animales de control ^5^, indican que el hipocampo es una estructura capaz de sufrir modificaciones funcionales por lesiones periféricas. De hecho, así quedó demostrado hace algunos años con lesiones del nervio ciático ^28^. Estas modificaciones funcionales, además de los cambios morfológicos caracterizados en las células piramidales de CA1 y CA3 asociados con la lesión del nervio facial (Trancoso J. Deterioro de la memoria espacial y modificaciones funcionales y estructurales en el hipocampo de ratas sometidas a lesión del nervio facial. Memorias, XII Congreso Nacional - XIII Seminario Internacional de Neurociencias 2021. https://colne.org.co/congreso/#speakers), podrían estar mediadas por las alteraciones en la microglía circundante observadas en el presente estudio. De hecho, existe evidencia de que las lesiones de nervios periféricos que producen dolor crónico, por ejemplo, provocan activación de la glia (microglía y astrocitos) y de citocinas proinflamatorias, lo que contribuye a la remodelación estructural del hipocampo y las funciones cognitivas que le subyacen ^29^^-^^32^.

La axotomía del nervio facial provoca una parálisis de las vibrisas que conduce a una modificación del procesamiento de información sensorio-motor, debido a la alteración de la adquisición activa de información sensorial. Si bien es conocido que el hipocampo no tiene conexión directa con las motoneuronas faciales lesionadas, se sabe que la corteza vM1 envía información al núcleo *reuniens*^33^ y que este, a su vez, proyecta sobre las células piramidales de CA1 del hipocampo ^34^. Entonces, la pasividad inducida por la axotomía del nervio facial de las entradas táctiles de las vibrisas, puede causar un desequilibrio crítico en este sistema, no solo por la degradación de las entradas del trigémino a la vM1 y al núcleo *reuniens*, sino también, por la modificación fisiológica y estructural de las neuronas piramidales de la capa 5 de la vM1, como se ha observado en trabajos previos ^2^^-^^4^.

El hipocampo es una estructura muy sensible a eventos estresantes. De hecho, se ha informado que el estrés agudo o crónico produce una activación de la microglía en varias estructuras del sistema nervioso central, incluido el hipocampo ^35^. Se ha observado que, tras lesiones del nervio facial, se produce un aumento significativo de los niveles plasmáticos de corticosterona ^7^, lo que podría relacionarse con el aumento de la proliferación y activación de la microglía descritos en este estudio.

Por último, se sabe que la microglía es capaz de remodelar estructuralmente las neuronas y, por lo tanto, modificar su función. Así que los hallazgos descritos aquí podrían explicar, al menos en parte, la disminución en la potenciación a largo plazo, las modificaciones morfológicas neuronales en las células CA3 a CA1 y los impedimentos en la consolidación de tareas dependientes del hipocampo observados previamente ^5^^,^^7^ (Troncoso J. Deterioro de la memoria espacial y modificaciones funcionales y estructurales en el hipocampo de ratas sometidas a lesión del nervio facial. Memorias, XII Congreso Nacional - XIII Seminario Internacional de Neurociencias 2021. https://colne.org.co/congreso/#speakers).
